# Quality of Spine Surgery Research from the Arab Countries: A Systematic Review and Bibliometric Analysis

**DOI:** 10.1155/2017/7560236

**Published:** 2017-02-21

**Authors:** Saleh S. Baeesa, Yazid Maghrabi, Abdul Karim Msaddi, Richard Assaker

**Affiliations:** ^1^Division of Neurosurgery, Department of Surgery, Faculty of Medicine, King Abdulaziz University, Jeddah, Saudi Arabia; ^2^Department of Neurosurgery, Neuro Spinal Hospital, Dubai, UAE; ^3^Department of Neurosurgery, Centre Hospitalier Regional Universitaire, 59000 Lille, France

## Abstract

*Purpose.* The purpose of our study is to evaluate the level of evidence (LOE) of spine surgery publications in the Arab countries and compare it with standard international literature in spine surgery and to determine the stand of the Arab nations academic production with that of the global one.* Methods.* An online search using “PubMed” and “Google Scholar” was carried out, using search terms related to spine surgery such as “Spine surgery,” “Scoliosis,” “Herniated disc.” Each article was reviewed and graded by two reviewers using Oxford Centre for Evidence-Based Medicine (OCEBM) Levels of Evidence scale.* Results.* We have identified 434 articles that met the inclusion criteria; 56% were level IV studies. The most common study design was case reports (42%). The number of Arab countries with publications in spine surgery was 18 countries. The country with the highest rate of publications was Egypt (26%). The quantity of the published studies increased from 151 in (2000–2008) to 283 in (2009–2015). There is statistical significance between high and low LOE articles (*p* = 0.0007).* Conclusion.* We have observed that LOE has not changed significantly over the period of 15 years and that much of the publications are of a low LOE (levels III and IV). We, herein, emphasize the need for spine surgeons in the Arab countries to conduct studies of higher LOE.

## 1. Introduction

With the current huge advances in information technology, the large quantity of medical information has exploded, posing a challenge to medical professional of how is the proper way of integrating this information into clinical practice [1]. Moreover, the need for a fundamental process that enables clinicians to assess and incorporate information drawn from scientific research has grown, leading to the development of evidence-based medicine (EBM), which became essential in the assessment of the quality of the published studies [2]. To our knowledge, there has been no study that quantifies the Arabic publications in spine surgery and determines the level of evidence (LOE) for it. We believe that LOE of Arabic publications in spine surgery is not well developed to the standard level in international publications due to several factors that will be mentioned later.

The aim of our study is to evaluate LOE of spine surgery publications in the Arab world and compare it with standard international literature in spine surgery and other specialties, to determine the stand of the Arab world academic production with that of the global one.

## 2. Materials and Methods

### 2.1. Search Strategy

This study was conducted in Jeddah, Saudi Arabia, between August and November 2015. A search strategy was developed for the retrieval of all spine surgery-related articles. This study was performed by accessing databases and using the following protocol: “Search term” AND “Country name.” The time interval was restricted to 1/1/2000–30/6/2015. Abstracts were screened, and if all inclusion criteria were met, then the full-text was accessed for more data.

### 2.2. Inclusion/Exclusion Criteria

Eligibility criteria for this study were all spine surgery-related clinical articles published in English or French with an abstract written in English in the time interval between January 2000 and June 2015. Moreover, the first author must be affiliated with an institution located in an Arabic country, and the populations of these studies, which being reviewed or recruited, must be in an organization based in an Arabic country. The exclusion criteria were all articles that dealt with animal studies, cadaveric studies, basic science, reviews, and editorials. Moreover, clinical studies that were published earlier than January 2000 were also excluded.

### 2.3. Information Sources

Systemic search using both “PubMed” and “Google Scholar” was carried out, using search terms related to spine surgery shown as follows: Spinal fusion, Spinal fixation, Spine surgery, Herniated disc, Discectomy, Laminectomy, Spinal cord. Each article retrieved by the manual research was reviewed by two reviewers and graded using Oxford Centre for Evidence-Based Medicine (OCEBM) Levels of Evidence Scale [3]. A point worth mentioning is that LOE grading was done after accessing the full-text of abstracts that met all the inclusion criteria; in other words, we relied on articles rather than abstracts.

### 2.4. Study Selection Process

After the complete review process, studies published in English or French with English abstracts, spine surgery-related, published between January 2000 and June 2015, with the first author being affiliated with an Arabic institution, were included in the analysis.

### 2.5. Data Items and Data Collection Process

Several items were collected from each article included in the analysis, namely, journal name, impact factor (IF), year of publication, affiliation, country, study design, LOE, citation numbers, and database. Regarding IF of individual journals, we collected 2015 IF for each journal. It is important to note that some articles were published in journals that were closed before 2015, so the corresponding IF for the last year of publication for such journals was included. Those items were collected in Excel spreadsheet. After the evaluation of each article, the result of the study was compared using several parameters similar to the ones published in another study [2], namely, Country, in other words, the country with the largest number of publications compared to the publications of the rest of countries. Moreover, publications in the time interval between 2000 and 2008 were compared to publications in the time interval between 2009 and 2015; papers published in journals with high impact factor (IF) were compared with publications published in other journals with low or no IF; research with high LOE level (levels I and II) was compared to research with LOE low level (levels III, IV, and V). Comparison of the result of this study with other local and international studies was done.

### 2.6. Statistical Analysis

Microsoft Excel (Microsoft, Redmond, Washington, USA) was used for the statistical analysis. Measures of central tendency such as mean and median were used for most parameters, along with parentage. Every single pair of data in this study was compared using *F*-test. A *p* < 0.05 and confidence interval of 95% were considered statistically significant. Kappa score was calculated to determine the degree of agreement between the two reviewers.

### 2.7. Review Reporting Style

This systematic review was reported in accordance with PRISMA statement [4].

## 3. Results

**Figure 1:**
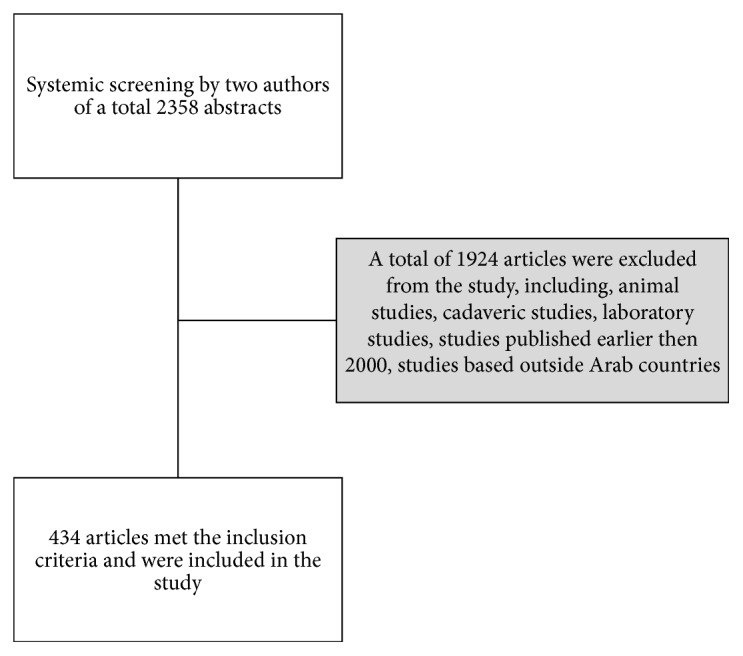
Schematic representation of the review process.

**Figure 2:**
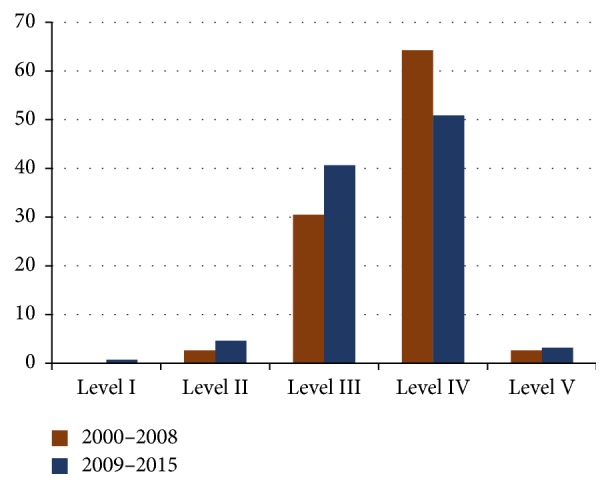
A graphic demonstration of the percentage of published studies and their level of evidence between January 2000 and December 2008 in comparison with studies published between January 2009 and June 2015.

Out of 2358 abstracts screened, only 434 articles that were published during 2000–2015 met the inclusion criteria of this study. The rest were excluded due to failure to meet the eligibility criteria of this study (Figure 1). The strength of agreement between the two reviewers was exquisite (Kappa = 0.908). The LOE of the articles in this study is as follows: 0.46% level I, 3.92% level II, 37.1% level III, 55.53% level IV, and 2.99% level V (Figure 2). The most commonly encountered study design in the data of our study was case reports: 181 (41.71%), followed by prospective studies: 114 (26.27%), retrospective studies: 76 (17.51%), case-series: 39 (8.99%), randomized controlled trials (RCT): 11 (2.53%), cross-sectional studies: 6 (1.38%), systemic review: 4 (0.92%), and case-control: 3 (0.69%).

**Figure 3:**
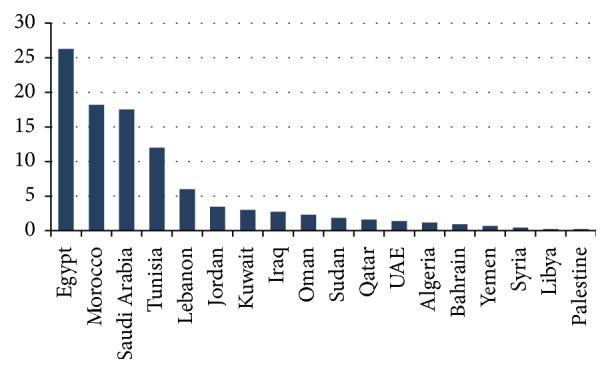
A graphic demonstration of the percentage of Arab countries contributions to spine surgery publications from January 2000 to June 2015.

There were 18 Arab countries with publications in spine surgery (Figure 3). The number of publications from individual countries was as follows: Egypt: 114 (26.27%), Morocco: 79 (18.20%), Saudi Arabia: 76 (17.51%), Tunisia: 52 (11.98%), Lebanon: 26 (6.00%), Jordan: 15 (3,46%), Kuwait: 13 (3.00%), Iraq: 12 (2.76%), Oman: 10 (2.30%), Sudan: 8 (1.84%), Qatar: 7 (1.61%), United Arab Emirates (UAE): 6 (1.38%), Algeria: 5 (1,15%), Bahrain: 4 (0.92%), Yemen: 3 (0.69%), Syria: 2 (0.46%), Libya: 1 (0.23%), and Palestine: 1 (0.23%).

IF of journals in this study ranged within 0.089–6.87 (median 1.426). 203 articles (29%) were published in journals with unrecorded IF. The number of journals used for publication was 143. The most frequently used 10 journals were as follows: Pan Arab Journal of Neurosurgery: 41 (9.45%), European Spine Journal: 19 (4.38%), The Spine Journal: 19 (4.38%), Neurosciences (Riyadh): 14 (3.23%), Egyptian Journal of Neurology, Psychiatry and Neurosurgery: 12 (2.76%), Asian Spine Journal: 11 (2.53%), Egyptian Journal of Neurosurgery: 11 (2.53%), International Orthopedics: 11 (2.53%), Joint Bone Spine: 11 (2.53%), and the Saudi Medical Journal: 11 (2.53%). The remaining 274 articles (63.13%) were published in 133 different journals.

The vast majority of the articles included in this study (91%) dealt with an adult population, whereas 8.99% dealt with pediatric cases. Papers written in English constituted 95.62% (415) of the total number of articles, whereas French papers constituted 4.4% (19).

Articles' citation numbers ranged within 0–136 (median 2); there were 176 (40.6%) articles with no recorded citation numbers. Moreover, the correlation coefficient between LOE and citation numbers was 0.12 (*p* = 0.0018), whereas the correlation coefficient between journals IF and citation numbers was 0.53 (*p* = 0.00004); Table 1 summarizes and compares different features of the results of this study.

## 4. Discussion

**Figure 4:**
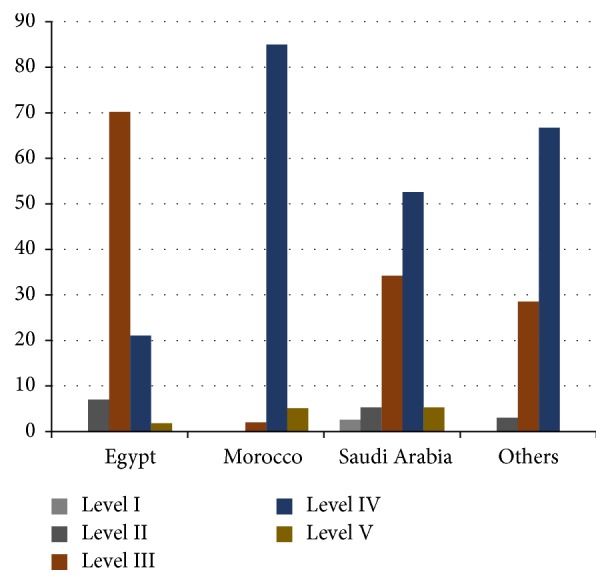
A graphic display of the percentage of Arab countries contributions to spine surgery research from January 2000 to June 2015 according to LOE.

The main aim of conducting this study was to evaluate the level and type of evidence of spine surgery publications published by Arabic institutions (Figure 4). After the application of this study's inclusion criteria, case reports, which provide a weak level of evidence, were included since they account for 41.7% of all publications [1]. Low-level studies (level III, level IV, and level V) accounted for 37.1%, 55.5%, and 2.99%, respectively (*p* = 0.0007). Moreover, high-level studies (either level I or level II) were 0.5% and 4%, respectively (*p* = 0.0007). Comparing the results of our study to several Saudi studies, which dealt with the LOE of Saudi publications in the areas of neurosurgery and orthopedics, we found that level IV papers constituted the vast majority of these studies, which is consistent with the results obtained from our data [2, 5].

Looking at citation numbers and their relationship with LOE, one can assume that articles with high LOE would have high citation numbers, but the result in our study proved that there is a weak negative correlation between the two (*r* = −0.12) (*p* = 0.0018). On the other hand, there was a somewhat good positive correlation between journals' IF and citation number (*r* = 0.53) (*p* = 0.00004).

Regarding IF of individual journals, as mentioned before in the method section, we included only IF of 2015 for each journal, and, in some cases, IF of years before 2015 were used in case if individual journals stop publishing in any year before 2015. One might argue that would there be a massive change of IF in the period included in this study? The answer is that many articles included in this review were published in journals not indexed in Thomson Reuters, so the information about IF of journals and their detailed analysis in the form of yearly impact, 5-year impact, and article influence status are unknown.

Regarding the tabulation of different parameters to evaluate the statistical significance, we found that statistical significance appeared when comparing the LOE of Egyptian publication, which is the country with the highest number of publications, with the rest of the countries, and also when comparing high-level LOE (I and II) with low-level LOE (III, IV, and V) (*p* = 0.014, *p* = 0.0007 resp.) (Table 1). The other parameters, such as time intervals and IF, proved no statistical significance (*p* = 0.18, *p* = 0.08, resp.) (Table 1).

Comparing the results to international literature, such as the study published by Wupperman et al. [6], it was found that most common literature published in the journals of Spine, American Journal of Sports Medicine, and Journal of Bone and Joint Surgery composed mainly of low-level studies (level IV, 53.60%, 42.90%, and 56.60%, resp.), bearing in mind that the scale used in Wupperman et al. is different from the one used in this study [6]. Moreover, it provides some consistency with the results of our study, in a way that most of the publications in these journals are of low-level evidence. Another study that was done by Amiri et al. [7] found that of 703 articles published in five different general spine journals, 59.6% of which were of level IV evidence, which in turn is consistent with our result.

The most frequent study type was case reports, which accounts for 41.71%, which is in conformity with the results published by other Saudi studies [2, 5]. These findings give some sort of consistency that most of the publications of surgical specialties in Arab countries and of the international literature are of low level of evidence, giving the fact that the proportion of systemic reviews of RCT and RCT itself, which constitutes the highest level of evidence, is low compared to the other types of studies. Furthermore, this can be tied to many factors, namely, lack of proper official training in research methodology, lack of time devoted to research, lack of interest in the research itself, lack of logistic support, and lack of financial assistance [2, 5, 8].

Ethical issues can also be of a high impact in conducting RCT. Poolman et al. argue that readers should not be misled by studies that are designated as level I or II to be of high quality since other methodological considerations must be evaluated and precautions must be taken [9].

To solve such issues and to increase the rate of publications, we have to deal with the issues from two sides: the side of the medical professionals and the side of the public. Regarding medical professionals, the concept of research and EBM should be introduced as early as from faculty of medicine, giving an early exposure to idea of the importance of such a concept [9]. Training the residents on research methodology and motivating and offering compensations for such acts would affect the quality and rate of publications considerably [10]. Senior physicians (surgeons) should be encouraged to do systemic reviews and meta-analysis of randomized trials, since they are not costly and less time-consuming, and provide nearly the highest level of evidence. Lastly, the public should be educated about the importance and the impact of clinical trials to help the community in providing interventions that are suitable for them [10]. Moreover, Koutras et al. argue that English proficiency play an important role in the quality and quantity of research output in non-English speaking countries, so focusing on encouraging medical students to be more proficient in English is a must, in order to solve the issue of poor quality and quantity of research output among Arab countries [11].

## 5. Limitations

We think that this study has a limitation regarding the interpretation of the results since most of the similar articles focus on either the publications of specific journals or publications about the particular geographical area, making the comparison biased. In-depth details of IF of journals such as yearly IF, 5-year impact, and article influence would have been reliable indicators of the quality of articles, but this was limited in this study, since a significant number of publications published in journals are not indexed in Thomson Reuters. Moreover, some studies might have been missed since we have restricted our search to only two databases “PubMed” and “Google Scholar”; these are major databases that usually include the majority of orthopedics, neurosurgery, and spine journals. We are aware that few journals may be overlooked because they are not found in our search. Moreover, this study restricted to limited languages (English and French).

## 6. Conclusions

In conclusion, spine diseases and its related procedures are not uncommon and rapidly growing in the Arab countries. There is general agreement that the current publications in the literature represent small numbers of research and have lower LOE and citations. We encourage our colleagues in the region to collaborate and produce larger studies, particularly of class I, with higher LOE.

## Figures and Tables

**Table 1:** Comparison of different parameters related to the collected data and their statistical significance.

Feature	Articles number	LOE *n* (%)					LOE mean	CI 95%	*p* value
		I	II	III	IV	V			
*Country*									
Egypt	114	0	8 (7%)	80 (70.2%)	24 (21.1%)	2 (1.8%)	3.18	(−2.01–8.91)	0.014 (S)
Other	320	2 (0.63%)	9 (2.8%)	81 (25.3%)	217 (67.8%)	11 (3.44%)	3.7		
*Year*									
2000–2008	151	0	4 (2.65%)	46 (30.46%)	97 (64.24%)	4 (2.65%)	3.67	(1.19–6.01)	0.18 (NS)
2009–2015	283	2 (0.71%)	13 (4.60%)	115 (40.64%)	144 (50.89%)	9 (3.18%)	3.51		
*IF*									
≥1	146	2 (1.37%)	8 (5.48%)	52 (35.62%)	82 (56.16%)	2 (1.37%)	3.51	(0.18–6.94)	0.08 (NS)
<1	288	0	9 (3.13%)	109 (37.85%)	159 (55.21%)	11 (3.82%)	3.6		
*Study LOE*									
High level (I, II)	19	2 (10.5%)	17 (89.5%)	0	0	0	1.89	(−7.04–12.58)	0.0007 (S)
Low level (I, IV, V)	415	0	0	160 (38.6%)	242 (58.3%)	13 (3.13%)	3.65		

IF: impact factor; LOE: level of evidence; S: significant; NS: not significant.
